# Supplementary material for: The Effects of Yoga Exercise on Blood Pressure and Hand Grip Strength in Chronic Stroke Patients: A Pilot Controlled Study
Source: Int J Environ Res Public Health. 2023 Jan 8;20(2):1108. doi: 10.3390/ijerph20021108 (PMC9859542; doi:10.3390/ijerph20021108)
Supplement: Supplementary file 1 [file ijerph-20-01108-s001.zip › ijerph-2091836-supplementary.pdf]

**Supplementary Materials:**

**File S1. Yoga Class schedule (12 Basic Yoga Pose)**

1. All session begins with warm-up(10 minutes) and ends with cold-down(10 minutes): Corpse Pose, mindfulness meditation, deeper/rhythmic breathing practice(inspiration/expiration time around 1:2).

2. Main activities(40 minutes): The yoga teacher who chooses 8-11 pose from the following 「12 Basic Yoga Pose」. In each class, each yoga pose is held for 10-60 seconds followed by 10-20 seconds of rest. Perform each yoga pose 3 to 5 times.

| Position | Photo | Yoga Pose | Number |
|---|---|---|---|
| Supine | 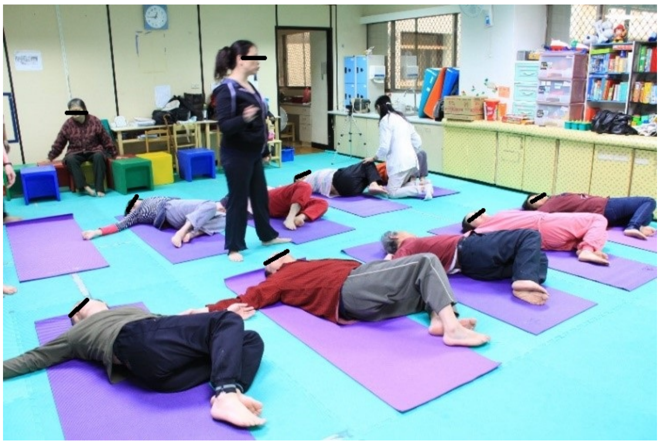 | supine spinal twist pose | 1 |
| | 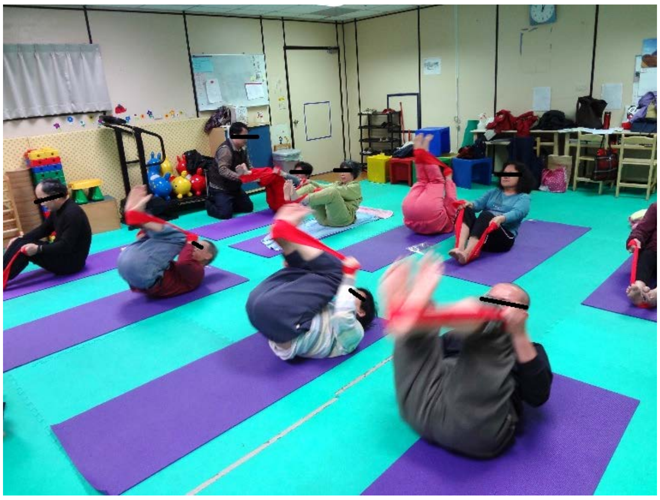 | knee to chest pose with/without integrating resistance bands | 2 |

| | | | |
|---|---|---|---|
| | 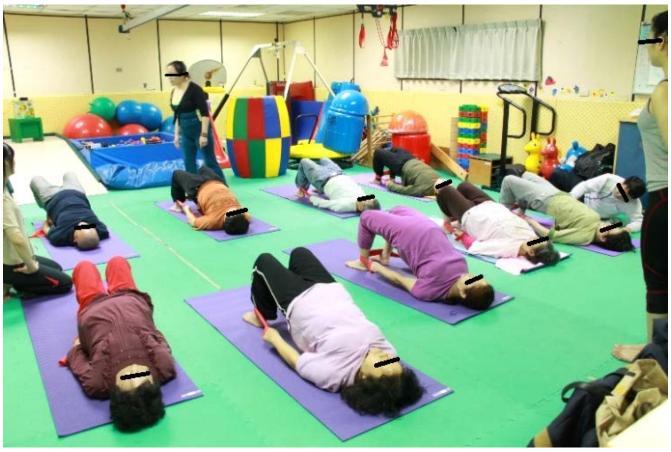 | bridge pose with/without integrating resistance bands | 3 |
| Prone | 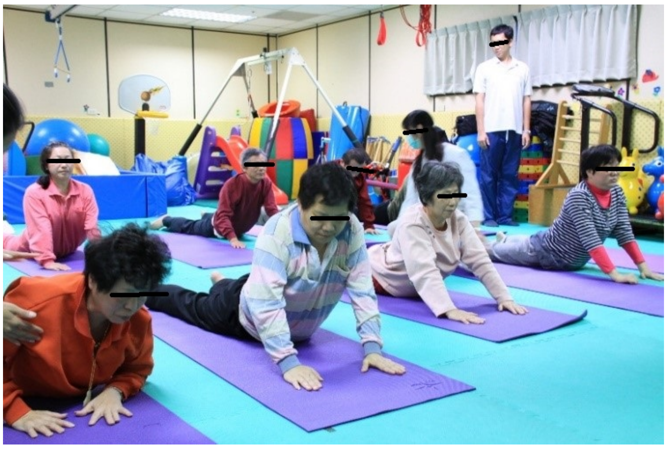 | cobra pose | 4 |
| Mat exercise | 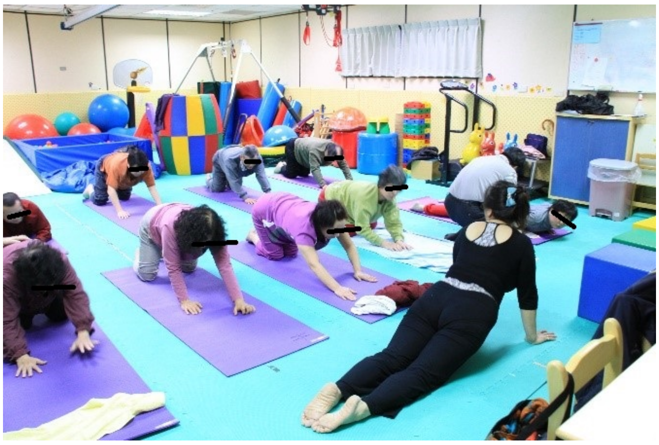 | cat pose | 5 |

| | | | |
|---|---|---|---|
| | 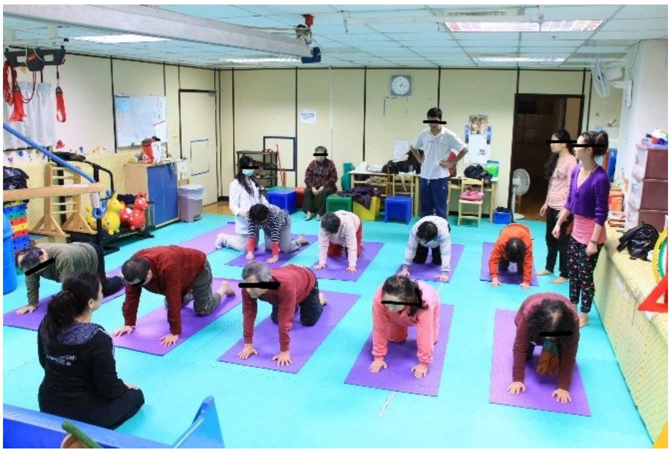 | cow pose | 6 |
| | 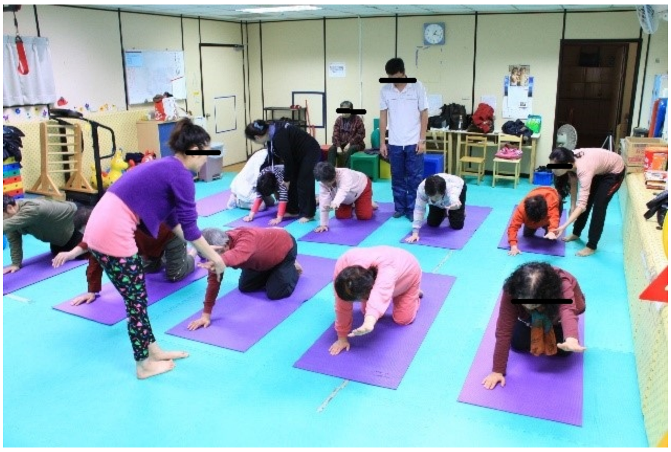 | modified balancing table pose | 7 |
| Seated | 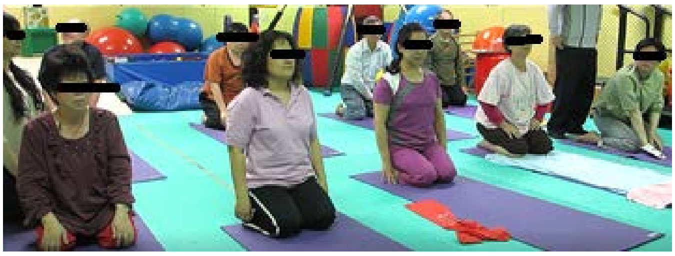 | hero pose | 8 |
| | 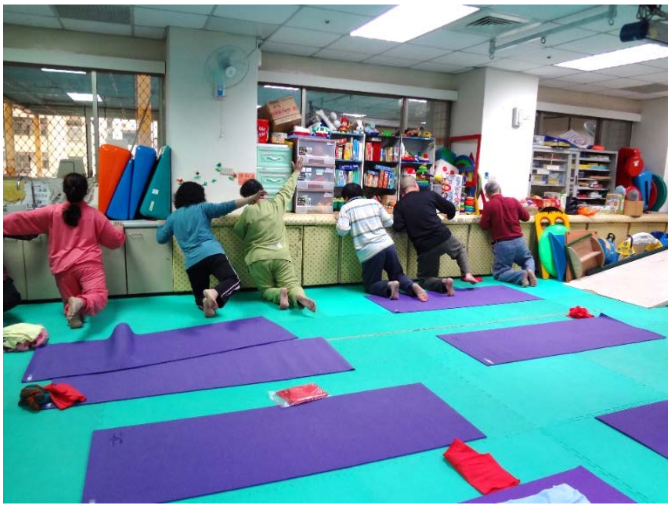 | kneeling pose with balancing on one knee | 9 |

| | | | |
|---|---|---|---|
| | 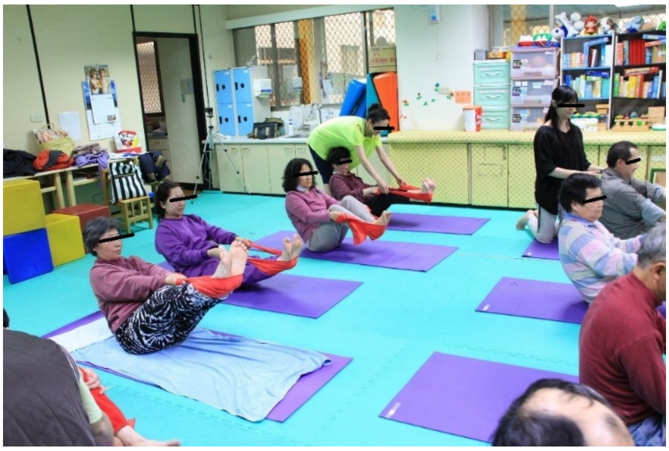 | modified boat pose with integrating resistance bands | 10 |
| | 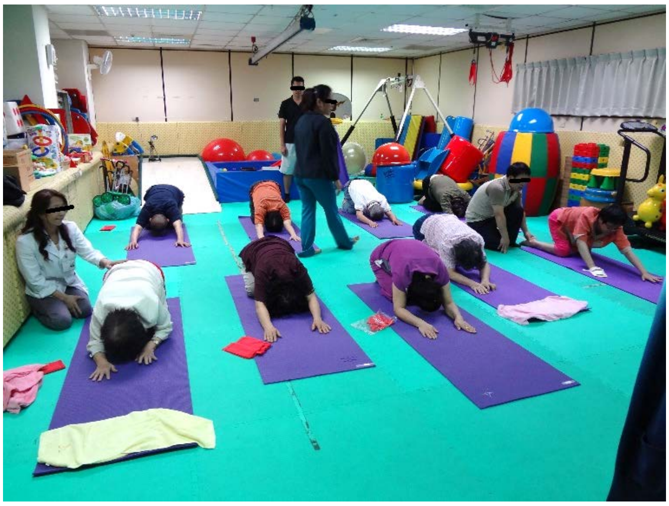 | hare pose | 11 |
| Standing | 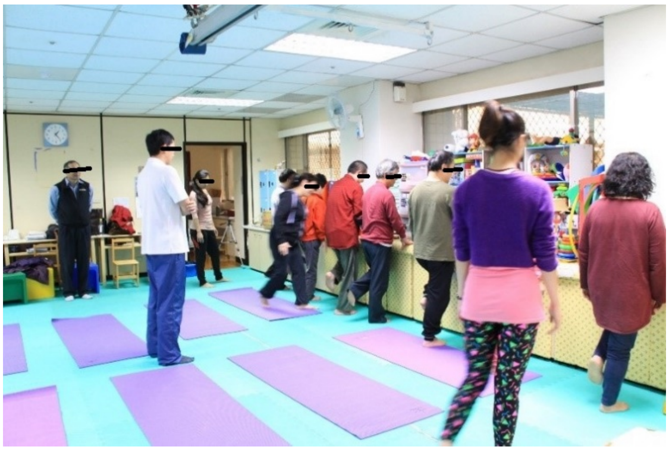 | modified tree pose | 12 |

**File S2. Standard stroke rehabilitation**

2. Frequency: 3 sessions in each weak
3. Time: 60 minutes in each session.
4. Training methods included

   Resistance exercise: weight-bearing training to increase the muscle strength of the lower limbs, such as repeated sit-to-stand training, or the use of equipment to increase the muscle strength of the lower limbs, such as progressive resistance training machines and stationary bicycles.

   Repetitive task-oriented training (include dynamic balance exercises and weight-shift training): sit-to-stand training, transfer, ambulation and going up and down stairs training.

   The physiotherapist who chooses 3-4 types of exercises from the following types of exercise. Perform each repeat each types of exercise 15 to 20 minutes.

| Types | Photo |
|---|---|
| sit-to-stand training | 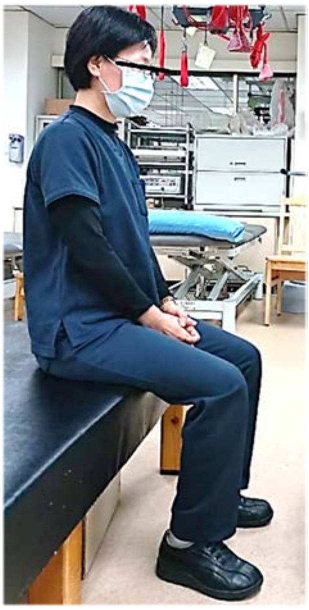 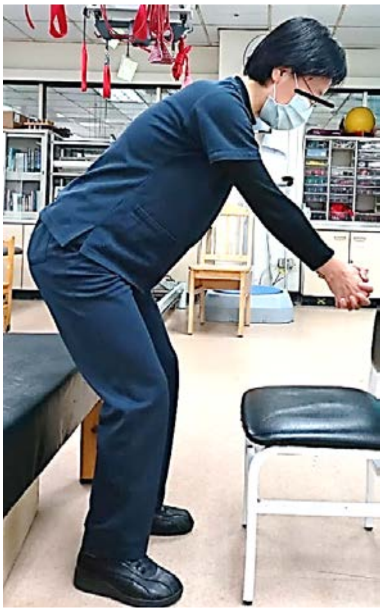 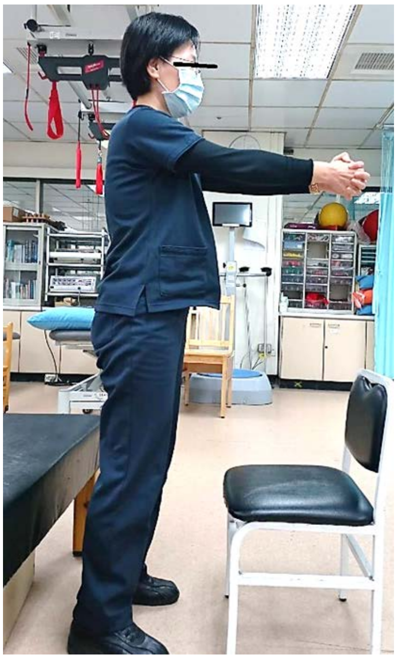 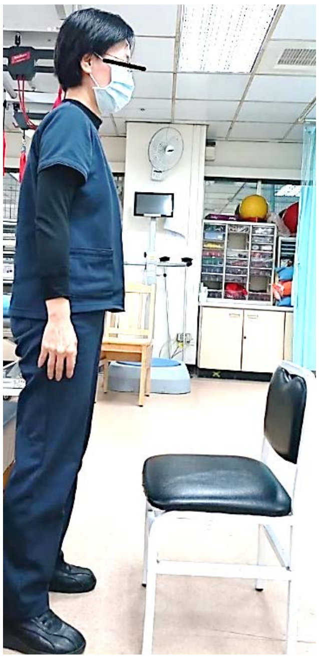 |

| | |
|---|---|
| progressive resistance training machines | 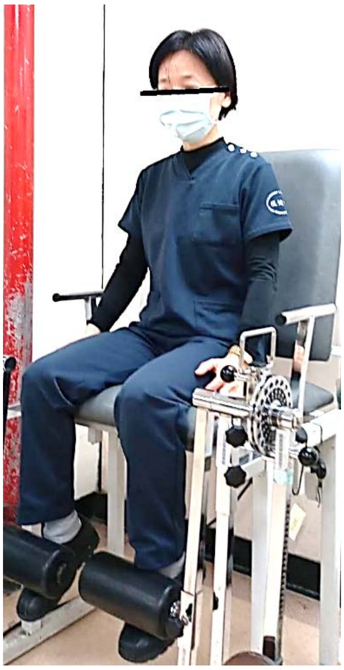 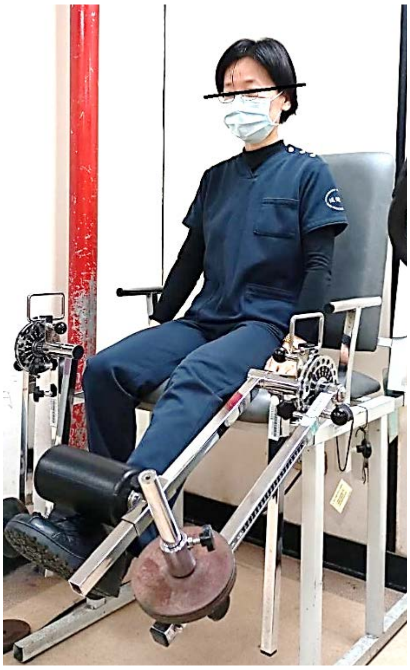 |
| stationary bicycles | 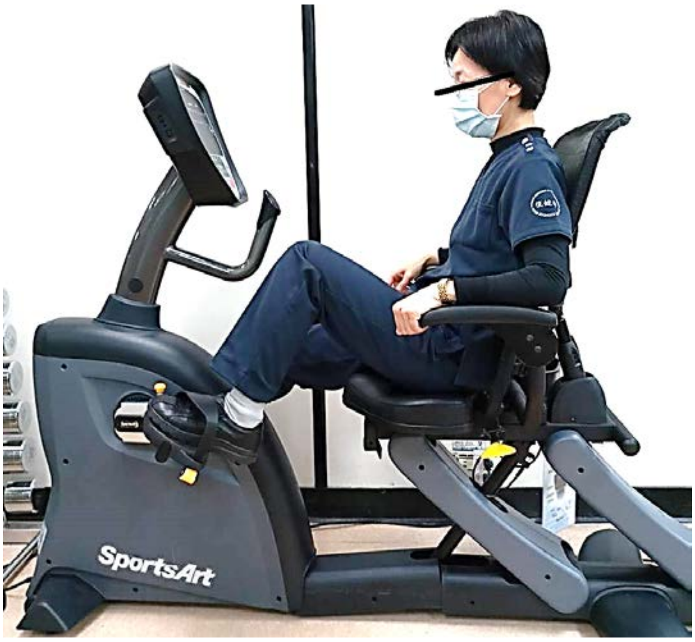  |

| | |
|---|---|
| ambulation | 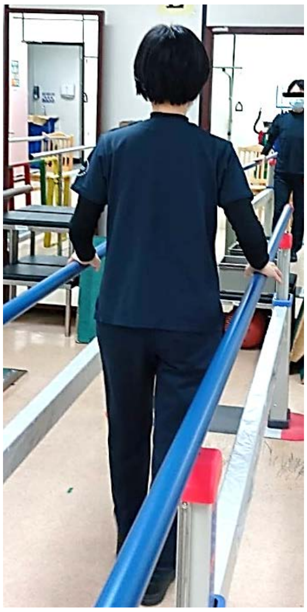 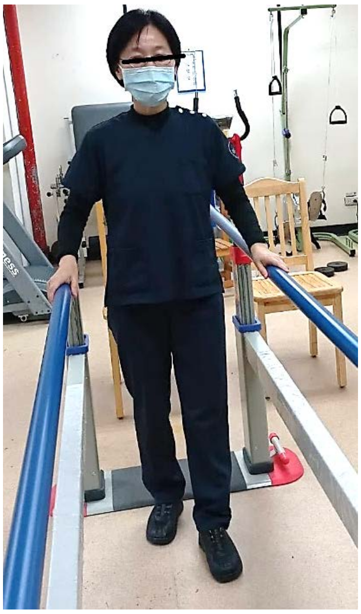 |
| | 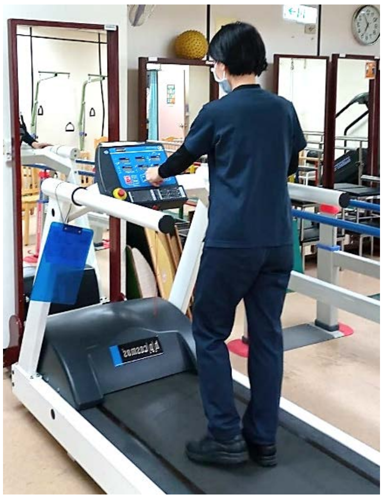 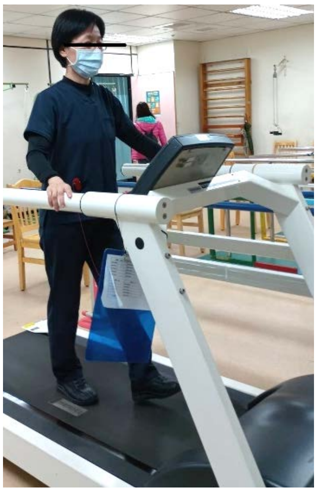 |
| going up and down stairs | 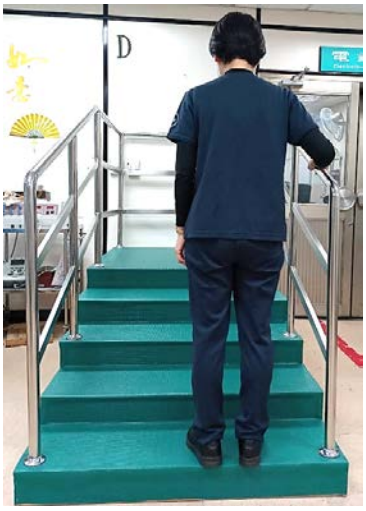 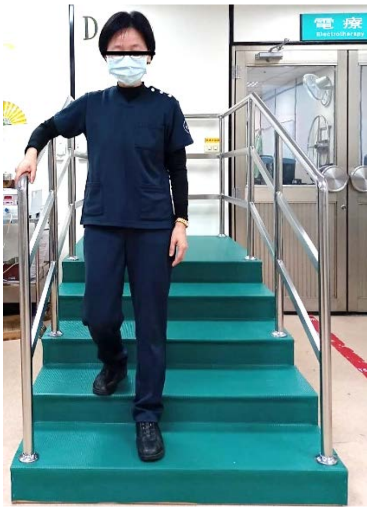 |

**File S3. Hand grip strength test**

Grip strength was measured by a handheld dynamometer (TTM-YD, Tokyo, Japan) and protocol as below.

#1.The tester adjusts the grip of the grip dynamometer so that when the subject's "hand" holds the grip, its proximal interphalangeal (pip) joint forms a right angle.

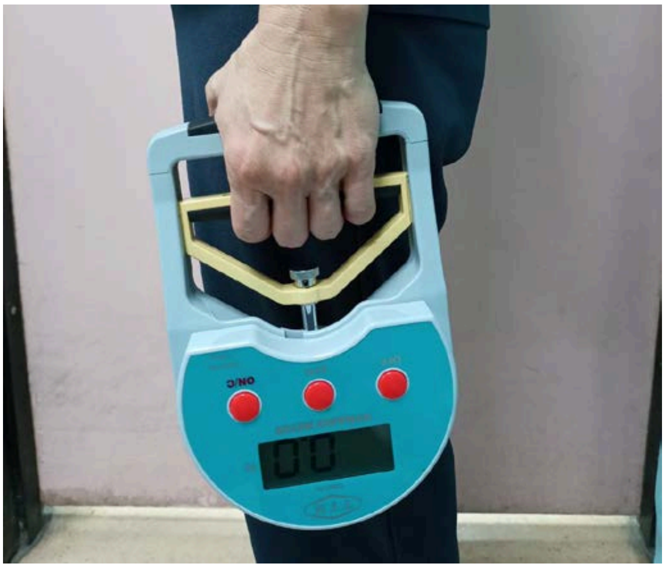

#2. The subjects stretched their arms and drooped naturally, looked straight ahead with both eyes, and paid attention that the grip dynamometer did not touch the body.

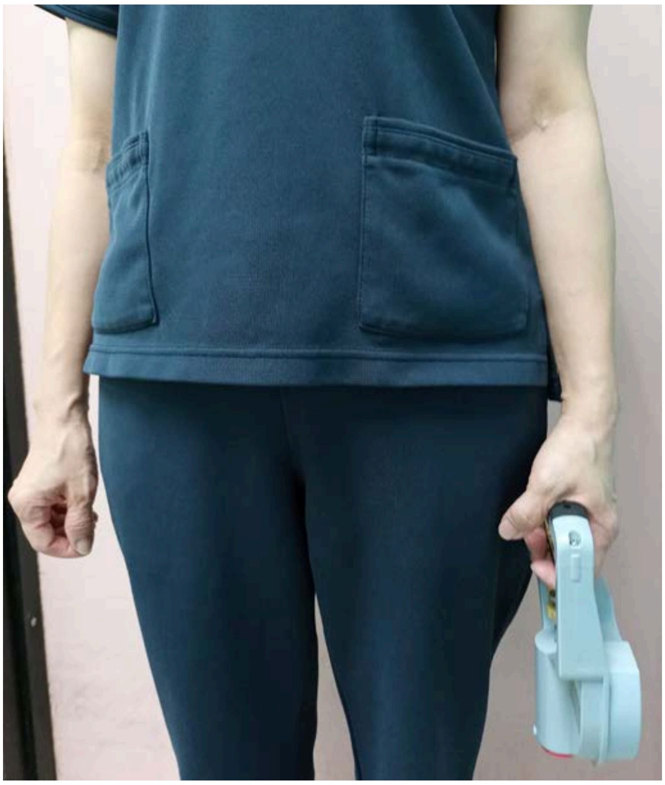

#3. According to this, a total of two measurements should be taken, and there should

be an appropriate rest for about one minute between the two measurements.
#4. Recording method: in kilograms, read up to 0.1 kilograms
